# Auraptene Has Antiviral Activity against Human Coronavirus OC43 in MRC-5 Cells

**DOI:** 10.3390/nu15132960

**Published:** 2023-06-29

**Authors:** Jung Sun Min, Young-Hee Jin, Sunoh Kwon

**Affiliations:** 1 KM Convergence Research Division, Korea Institute of Oriental Medicine, Daejeon 34054, Republic of Korea; jsmin1019@kiom.re.kr; 2 KM Application Center, Korea Institute of Oriental Medicine, Daegu 41062, Republic of Korea

**Keywords:** HCoV-OC43, auraptene, human coronavirus, MRC-5, antiviral, matrix metalloproteinase, MMP-9, MMP-2, *Citrus trifoliata*

## Abstract

Auraptene (7-geranyloxycoumarin) is the abundant prenyloxycoumarin found in the fruits of *Citrus* spp. Auraptene has a variety of pharmacological and therapeutic functions, such as anticancer, antioxidant, immunomodulatory, and anti-inflammation activities, with excellent safety profiles. In this study, we evaluated the anticoronaviral activity of auraptene in HCoV-OC43-infected human lung fibroblast MRC-5 cells. We found that auraptene effectively inhibited HCoV-OC43-induced cytopathic effects with 4.3 μM IC_50_ and 6.1 μM IC_90_, resulting in a selectivity index (CC_50_/IC_50_) of >3.5. Auraptene treatment also decreased viral RNA levels in HCoV-OC43-infected cells, as detected through quantitative real-time PCR, and decreased the expression level of spike proteins and nucleocapsid proteins in virus-infected cells, as detected through the Western blot analysis and immunofluorescence staining. Time-of-addition analysis showed auraptene’s inhibitory effects at the post-entry stage of the virus life cycle; however, auraptene did not induce the antiviral interferon families, IFN-α1, IFN-β1, and IFN-λ1. Additionally, auraptene-treated MRC-5 cells during HCoV-OC43 infection decreased the MMP-9 mRNA levels which are usually increased due to the infection, as auraptene is a previously reported MMP-9 inhibitor. Therefore, auraptene showed antiviral activity against HCoV-OC43 infection, and we suggest that auraptene has the potential to serve as a therapeutic agent against human coronavirus.

## 1. Introduction

Coronaviruses (CoVs) are the enveloped positive-sense single-stranded RNA viruses belonging to the *Nidovirales* order of the *Coronaviridae* family, which is divided into four genera, namely *Alphacoronavirus*, *Betacoronavirus*, *Gammacoronavirus*, and *Deltacoronavirus*, along with the seven strains of human infection CoVs [1]. Among the seven human coronaviruses (HCoVs), HCoV-NL63 and HCoV-229E are included in the *Alphacoronavirus* genus. Severe acute respiratory syndromes, (SARS)-CoV, and SARS-CoV-2, and Middle East respiratory syndrome (MERS)-CoV, as well as HCoV-OC43 and HCoV-HKU1, are included in the *Betacoronavirus* genus [2]. The coronavirus disease 2019 (COVID-19) pandemic, caused by SARS-CoV-2 infection, engendered a global health crisis that led to 770 million confirmed cases and 7 million deaths over three years as of the end of May 2023 [3]. Despite the development of several highly effective COVID-19 vaccines, many people are still vulnerable to virus infection and developing the disease due to the high rate of transmissibility and the frequent occurrence of concerning variants of SARS-CoV-2. The Food and Drug Administration approved Veklury^®^ (remdesivir) as the first antiviral treatment for COVID-19 and Lagevrio™ (molnupiravir) and Paxlovid™ (ritonavir-boosted nirmatrelvir) for emergency use authorization during the COVID-19 pandemic, but additional antiviral therapeutics to treat and prevent the disease are still needed. Among all CoVs, HCoV-OC43 was isolated in the 1960s and is the most prevalent HCoV globally [4]. HCoV-OC43 infection usually causes a common cold; however, it can progress to bronchitis or pneumonia, comprising around 10% of all respiratory tract infections [5]. Experimental models of HCoV-OC43 infection in human lung cells are useful for evaluating the biological and anticoronaviral activity of various candidate agents because they can be safely conducted in a biosafety level (BSL)-2 facility, unlike the highly pathogenic members of the *Betacoronavirus*, SARS-CoV, MERS-CoV, and SARS-CoV-2, the experimental models of which must be conducted in BSL-3 facilities [6,7].

Monoterpene coumarin ether, also known as auraptene (7-geranyloxycoumarin), was first discovered and characterized in *Citrus aurantium* L. (Rutaceae), known as bitter orange, in 1930 [8]. It is the best known prenyloxycoumarin and commonly found in the fruits of *Citrus* spp., such as oranges, lemons, and grapefruits [9]. A toxicity study of auraptene demonstrated its excellent safety profile, as it fell within normal ranges for histopathological, hematological, biochemical parameters. Furthermore, no mortality was observed. Auraptene exhibits a variety of pharmacological and therapeutic properties, such as anticancer, neuroprotective, cardioprotective, gastrointestinal protective, immunomodulatory, anti-inflammatory, antioxidant, antibacterial, antiprotozoal, and antifungal activities [10]. Notably, auraptene has shown anticancer activity against various types of cancer by targeting multiple cellular signaling pathways involved in proliferation, apoptosis, growth factors, cytokines, and transcription factors. Additionally, it inhibits the biological function of target proteins, such as matrix metalloproteinases (MMPs), peroxisome proliferator-activated receptors, and acetylcholinesterase [11,12]. Recently, auraptene was reported to have antiviral activity against the influenza A virus, H1N1 [13], as well as to inhibit enterovirus 71 infection at the attachment and entry steps of their life cycle, targeting the viral envelope proteins, VP1 and VP4 [14]. However, the anticoronaviral activity of auraptene remains unclear. Thus, we examined the anticoronaviral activity and mode of action of auraptene against HCoV-OC43 in the human lung cell line, MRC-5. We found that auraptene effectively inhibits HCoV-OC43 infection in MRC-5 cells, suggesting that it could be a potential anticoronaviral therapeutic agent.

## 2. Materials and Methods

### 2.1. Compound

Auraptene (PubChem CID: 1550607, and molecular weight: 298.4) isolated from the peel of *Poncirus trifoliata* (L.) Raf. (also known as *Citrus trifoliata*) was acquired from Wuhan ChemFaces Biochemical Co., Ltd. (Wuhan, China) and 20 mM stock solutions were created in dimethyl sulfoxide (DMSO) (Sigma-Aldrich, St. Louis, MO, USA) and stored at −80 °C in a deep freezer for long-term storage.

### 2.2. Cells and Virus

Human fetal lung fibroblast MRC-5 cells from the American Type Culture Collection (ATCC, Manassas, VA, USA) were cultured in minimal essential medium (Corning Incorporated, Corning, NY, USA, Cat. No. 10-009-CV) supplemented with 10% fetal bovine serum (Gibco, Carlsbad, CA, USA) and 1% penicillin/streptomycin (Gibco) at 37 °C in a 5% CO_2_ incubator. HCoV-OC43 (ATCC, Manassas, VA, USA) was propagated in MRC-5 cells at 33 °C in a 5% CO_2_ incubator and titrated, as previously described [15].

### 2.3. Cytopathic Effect Reduction Assay

MRC-5 cells were seeded at 1 × 10^4^ cells/96-well plate (Thermo Fisher Scientific Inc., Waltham, MA, USA) overnight. MRC-5 cells were infected with HCoV-OC43 (10^3.5^ TCID_50_/100 μL) and treated with the serially diluted compound at 33 °C incubator for 4 days. A colorimetric MTS [3-(4,5-dimethylthiazol-2-yl)-5-(3-carboxymethoxyphenyl)-2-(4-sulfophenyl)-2*H*-tetrazolium] assay kit (Promega Corporation, Madison, WI, USA) was used to quantify viable cells at 4 days post-infection following the manufacturer’s instructions. Then, absorbance at 490 nm was measured using a GloMax^®^ Microplate Reader (GM3000, Promega). The absorbance of vehicle-treated cells was set as 100% viable cells and the absorbance of infected and vehicle-treated cells was set as 0% viable cells. Final DMSO concentration was maintained at <0.1%.

### 2.4. HCoV-OC43 Viral RNA Copy Number Analysis

Viral RNAs were harvested from the culture media supernatant of HCoV-OC43-infected MRC-5 cells using a QIAamp viral RNA mini kit (Qiagen N.V., Hilden, Germany), and viral RNAs from cell lysate of HCoV-OC43-infected MRC-5 cells were isolated using the RNeasy^®^ Mini Kit (Qiagen), following manufacturer’s instructions. These RNAs were used to synthesize and amplify cDNA using a One Step TB Green^®^ PrimeScript™ RT-PCR kit (Takara Bio Inc., Kusatsu, Japan) and CFX Opus 96 Real-time PCR system (Bio-Rad Lab., Hercules, CA, USA) according to manufacturer’s instructions with the following primers for the HCoV-OC43 Nucleocapsid protein: (sense primer) 5′-AGCAACCAGGCTGATGTCAATACC-3′ and (antisense primer) 5′-AGCAGACCTTCCTGAGCCTTCAAT. A standard curve of HCoV-OC43 viral RNA was used to calculate the viral RNA copy number, as previously described [15].

### 2.5. Western Blot Assay

Cells were lysed in Glo lysis buffer (Promega), total cell lysates were separated on a 10% SDS-PAGE gel (Bio-Rad Lab), and proteins were transferred to a PVDF membrane (Bio-Rad Lab). The transferred membranes were incubated with 5% skim milk in TBST (TBS with 0.5% Tween20) for 30 min at 25 °C and washed three times with cold TBST. Thereafter, the anti-HCoV-OC43 spike protein antibody (Catalog Number CSB-PA336163EA01HIY, Cusabio, Houston, TX, USA), anti-HCoV-OC43 nucleocapsid protein antibody (Cat. No. MAB9012, Merck & Co., Inc., Rahway, NJ, USA), or anti-β-actin antibody (Cat. No. 3700S, Cell Signaling Technology, Danvers, MA, USA) was incubated at 4 °C overnight. The membranes were then washed with cold TBST and interacted with secondary antibodies conjugated with horseradish peroxidase (Abcam PLC, Cambridge, UK) for 1 h at 25 °C. Protein bands were observed using a Clarity Max Western ECL substrate (Bio-Rad) and the Chemidoc™ MP Gel Imaging System (Bio-Rad).

### 2.6. Immunofluorescence Staining Assay

MRC-5 cells were grown on poly-L-lysine-coated coverslip, fixed with 4% paraformaldehyde for 10 min and rinsed with cold PBS. Then, cells were permeabilized in 0.2% Triton X-100-contained PBS for 10 min and blocked with 3% BSA in permeabilizing buffer. Cells were incubated with anti-HCoV-OC43 spike protein antibody (CusaBio), or anti-HCoV-OC43 nucleocapsid protein antibody (Merck) at 4 °C overnight. Cells were washed with cold PBS three times and incubated with AlexaFluor488-conjugated goat anti-rabbit IgG antibody (Cat. No. A11001, Thermo Fisher Scientific Inc.) or AlexaFluor555-conjugated goat anti-mouse IgG antibody (Cat. No. A21428, Thermo Fisher, Waltham, MA, USA) for 1 h at room temperature. These cells were washed with PBS and mounted using SlowFade™ Gold Antifade Mountant with DAPI (Invitrogen, Waltham, MA, USA) and visualized using an Olympus IX71 fluorescence microscope (Olympus Corporation, Tokyo, Japan) and the cellSens program (Olympus).

### 2.7. Time-of-Addition Assay

MRC-5 cells were grown overnight at a density of 1 × 10^4^ cells/96-well culture plate. As pretreatment, the cells were pretreated with the indicated concentration of auraptene for 4 h before HCoV-OC43 infection and then the compound was removed. Cells were infected with the virus and incubated for four days. For cotreatment, the compound was added with the virus for 4 h; then, cells were washed and incubated with the virus for four days. As posttreatment, cells were infected with the virus and after 4 h, the compound was added with the virus during four days. At 4 days postinfection, a cytopathic effect reduction assay was performed using an MTS assay kit (Promega).

### 2.8. Quantitative Real-Time PCR (qRT-PCR)

Total RNA of cell lysates was isolated using the RNeasy^®^ Mini kit (Qiagen) and used to synthesize and amplify cDNA via the TB Green^®^ PrimeScript^TM^ RT-PCR Kit (Takara Bio) and the CFX Opus 96 Real-time PCR system (Bio-Rad Lab). The following primers were used: for IFN-α1, 5′-GTGCTCAGCTGCAAGTCAAG-3′ and 5′-TATCCAGGCTGTGGGTCTC-3′; for IFN-β1, 5′-TAGCACTGGCTGGAATGAG-3′ and 5′-GTTTCGGAGGTAACCTGTAAG-3′; for IFN-λ1, 5′-GTCACCTTCAACCTCTTCCG-3′ and 5′-TCAGACACAGGTTCCCATCG-3′; for MMP-2, 5′-TCTCCTGACATTGACCTTGGC-3′ and 5′-CAAGGTGCTGGCTGAGTAGATC-3′; for MMP-9, 5′-TTGACAGCGACAAGAAGTGG-3′ and 5′-GCCATTCACGTCGTCCTTAT-3′; and for β-actin, 5′-GGAAATCGTGCGTGACATCA-3′ and 5′-ATCTCCTGCTCGAAGTCCAG-3′ [15,16].

### 2.9. Statistical Analysis

Statistical analysis was performed using GraphPad Prism Software V.9.5.1 purchased from GraphPad Software, Inc. (San Diego, CA, USA). Data are represented as means ± standard errors of the mean (SEMs). Probability (*p*) values were obtained through one-way or two-way ANOVA followed by Bonferroni’s test, as described in the figure legends. (* *p* < 0.05, ** *p* < 0.01, *** *p* < 0.001, and **** *p* < 0.0001).

## 3. Results

### 3.1. Auraptene Inhibits the HCoV-OC43-Induced Cytopathic Effects

**Figure 1:**
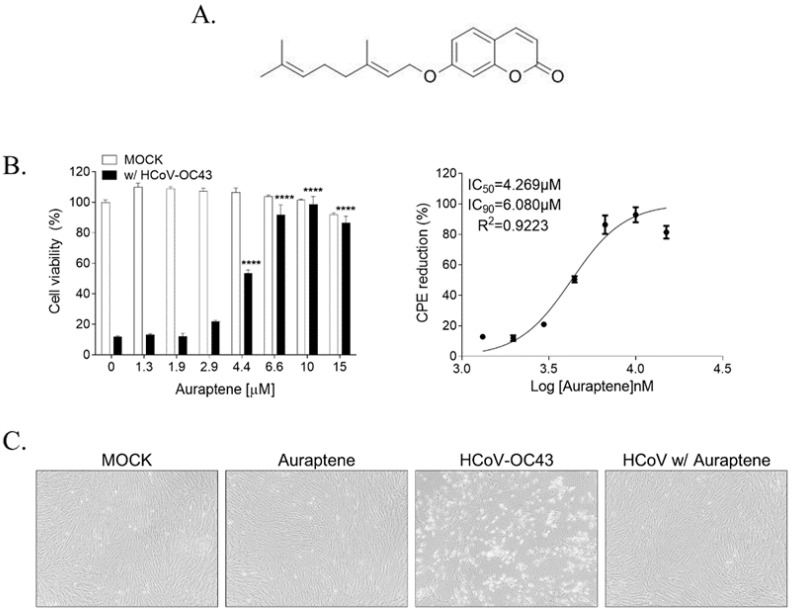
HCoV-OC43-induced cytopathic effects were inhibited by auraptene treatment in human lung fibroblast cells, MRC-5. (**A**) Chemical structure of auraptene. (**B**) MRC-5 cells were infected with 10^4.5^ TCID_50_/mL HCoV-OC43 and treated with serially diluted concentrations of auraptene from 1.3 to 15 μM. At 4 days postinfection, an MTS assay was conducted to detect cell viability (left graph). The IC_50_ and IC_90_ of auraptene were 4.3 μM and 6.1 μM, respectively, as calculated via nonlinear regression analysis (right graph). Data were analyzed using a one-way analysis of variance followed by Bonferroni’s multiple comparisons test (F_7,16_ = 120.5, *p* < 0.0001; **** *p* < 0.0001 vs. 0 μM of auraptene). (**C**) Optical microscope images of mock, 10 μM auraptene-treated cells, HCoV-OC43-infected cells, or virus-infected and 10 μM auraptene-treated cells at 4 days postinfection. Data are presented as mean ± SEM and represent three independent experiments.

To investigate the anticoronaviral activity of auraptene (Figure 1A), we examined its effect on HCoV-OC43-induced cytopathic effects in a human lung cell line, MRC-5 cells. Auraptene did not induce cytotoxic effects under 10 μM, although approximately 10% of cell death was induced by 15 μM of auraptene, suggesting 50% of cytotoxic concentration (CC_50_) of >15 μM. When MRC-5 cells were infected with 10^4.5^ TCID_50_/mL of HCoV-OC43, virus infection induced around 89% of cell death due to cytopathic effects at 4 days postinfection. However, treatment with serially diluted concentrations of auraptene reduced the HCoV-OC43-induced cytopathic effects dose-dependently (Figure 1B). The 50% and 90% inhibitory concentration (IC_50_) and (IC_90_) values were 4.3 μM and 6.1 μM, respectively, as obtained via nonlinear regression analysis, resulting in a selectivity index (CC_50_/IC_50_) of >3.5. Thus, we used up to 10 μM of auraptene for further experiments. In addition, we confirmed the complete protection of 10 μM auraptene from the HCoV-OC4-induced cytopathic effects based on cellular morphology. The data revealed that treatment with 10 μM auraptene effectively prevented cell death caused by HcoV-OC43-induced cytopathic effects (Figure 1C, the third image, HCoV-OC43). Thus, the morphology of virus-infected cells treated with auraptene (Figure 1C, the fourth image, HCoV w/ Auraptene) remained intact, resembling the morphology of non-infected cells (Figure 1C, the first image, MOCK) and auraptene-treated cells (Figure 1C, the second image, Auraptene). Therefore, these data showed that auraptene efficiently protected from HCoV-OC43-induced cytopathic effects.

### 3.2. Auraptene Inhibits HCoV-OC43 Viral RNA Replication and Viral RNA Expression

**Figure 2:**
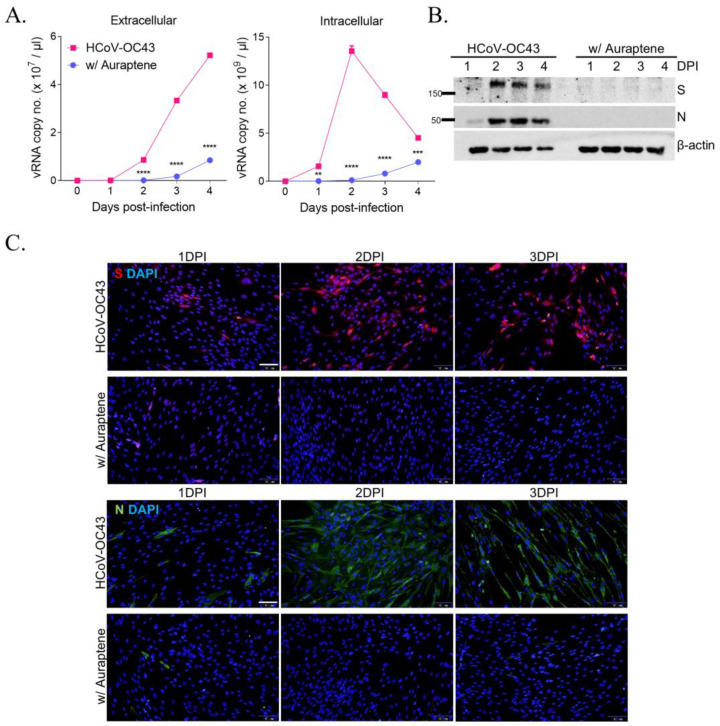
HCoV-OC43 replication and the expression of viral protein were inhibited by 10 μM auraptene treatment. (**A**) Extracellular (left graph) and intracellular viral RNA levels (right graph) in HCoV-OC43-infected MRC-5 cells treated with (circle) or without (rectangle) 10 μM auraptene assessed using qRT-PCR at 1, 2, 3, and 4 days postinfection. Data were analyzed using two-way ANOVA followed by Bonferroni’s multiple comparisons test (extracellular: time effect, F_4,8_ = 3050, *p* < 0.0001; auraptene effect, F_1,8_ = 5590, *p* < 0.0001; time × auraptene interaction, F_4,8_ = 1690, *p* < 0.0001; intracellular: time effect, F_4,8_ = 273.4, *p* < 0.0001; auraptene effect, F_1,8_ = 1126, *p* < 0.0001; time × auraptene interaction, F_4,8_ = 281.4, *p* < 0.0001; ** *p* < 0.01, *** *p* < 0.001, and **** *p* < 0.0001 vs. HCoV-OC43 at the same day postinfection). Data are presented as mean ± SEM. (**B**) Expression of viral spike (S) proteins and nucleocapsid (N) protein in MRC-5 cells with HCoV-OC43 infection and treatment of vehicle or 10 μM auraptene at 1, 2, 3, and 4 days postinfection according to the Western blot analysis. The β-actin band was detected as a loading control. Data are representative of three independent experiments. (**C**) Immunofluorescence staining images at 1, 2, and 3 days postinfection. HCoV-OC43 spike protein (red) and nucleus visualized with DAPI (blue) (upper panel), and N protein (green) and nucleus visualized with DAPI (blue) (lower panel) in MRC-5 cells virus-infected with vehicle or 10 μM auraptene treatment. Scale bar = 100 μm. The experiments were performed independently ≥3 times.

To examine the effect of auraptene on viral replication, intracellular and extracellular viral RNA levels were measured in HCoV-OC43-infected and 10 μM auraptene-treated MRC5 cells using qRT-PCR. The level of extracellular viral RNA increased up to 5 × 10^7^ viral RNA copy number/μL, however, auraptene treatment decreased it to 8 × 10^6^ viral RNA copy number/μL at 4 days postinfection. In addition, the intracellular viral RNA level peaked at 1.4 × 10^10^ viral RNA copy number/μL at 2 days postinfection, and decreased with auraptene treatment to 1.2 × 10^9^ viral RNA copy number/μL at 2 days postinfection (Figure 2A). Moreover, the Western blot analysis showed that viral proteins spike (S, 145 kDa) and nucleocapsid protein (N, 50.4 kDa) could be detected in HCoV-OC43-infected cells but not in 10 μM auraptene-treated MRC5 cells between 2 and 4 days postinfection (Figure 2B). Immunofluorescence staining analysis also showed that spike (S) and nucleocapsid protein (N) could be detected in HCoV-OC43-infected cells between 1 and 3 days postinfection but not in 10 μM auraptene-treated MRC5 cells (Figure 2C). Therefore, these data showed that auraptene inhibited viral replication and the expression level of viral proteins, suggesting the antiviral activity of auraptene against HCoV-OC43 infection.

### 3.3. Auraptene Inhibited HCoV-OC43 Infection at the Post-Entry Infection Stage

**Figure 3:**
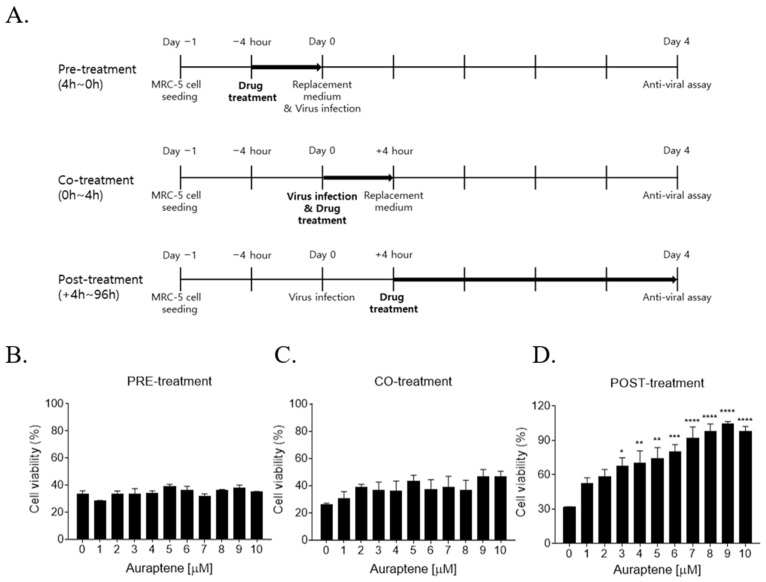
Time-of-addition assay of auraptene during HCoV-OC43 infection in MRC-5 cells. (**A**) Scheme for the time-of-addition assay. (**B**–**D**) Cell viability of pretreatment (**B**), cotreatment (**C**), and posttreatment (**D**) assay assessed using MTS at 4 days postinfection. Data were analyzed using a one-way analysis of variance (ANOVA) followed by Bonferroni’s multiple comparisons test (PRE-treatment: F_10,22_ = 1.978, *p* = 0.0877; CO-treatment: F_10,22_ = 1.232, *p* = 0.3251; POST-treatment: F_10,22_ = 10.61, *p* < 0.0001; * *p* < 0.05, ** *p* < 0.01, *** *p* < 0.001 and **** *p* < 0.0001 vs. 0 μM of auraptene). Data are presented as mean ± SEM and representative of three independent experiments.

To define the mechanism of auraptene’s antiviral effects, a time-of-addition assay was conducted (Figure 3A). In the pretreatment experiment, MRC-5 cells were pretreated with 10 μM auraptene for 4 h, washed, and then infected with HCoV-OC43 for 4 days. In the cotreatment experiment, MRC-5 cells were cotreated with 10 μM auraptene and HCoV-OC43 infection for 4 h. At 4 days postinfection, auraptene did not inhibit HCoV-OC43 infection based on cytopathic effects reduction data in the pre- and cotreatment experiments (Figure 3B,C). In the posttreatment assay, MRC-5 cells were infected with HCoV-OC43 and then treated with 10 μM auraptene at 4 h postinfection. Auraptene was found to inhibit virus-induced cytopathic effects in the posttreatment assay (Figure 3D). These data suggested that auraptene inhibited HCoV-OC43 infection after 4 h of infection, which corresponds to the post-entry stage.

### 3.4. Auraptene Did Not Induce the Antiviral IFN Gene Expression

**Figure 4:**
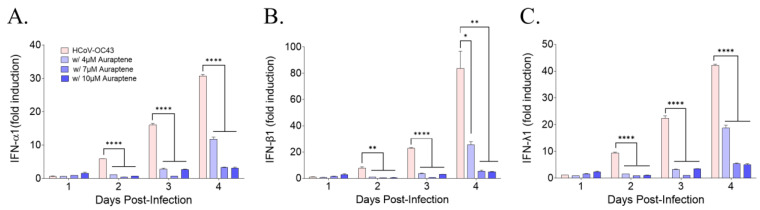
IFN-related antiviral response was not induced by auraptene treatment during HCoV-OC43 infection. (**A**–**C**) Level of IFN-α1 (**A**), IFN-β1 (**B**), and IFN-λ1 (**C**) mRNA induction quantified in HCoV-OC43-infected MRC-5 cells with vehicle (pink bar) or 4, 7, or 10 μM auraptene (blue bar) treatment using qRT-PCR at 1, 2, 3, and 4 days postinfection. Data were normalized to β-actin gene expression level. Data were analyzed using one-way ANOVA followed by Bonferroni’s multiple comparisons test (IFN-α1: 2 days postinfection, F_3,4_ = 18177, *p* < 0.0001; 3 days postinfection, F_3,4_ = 713.7, *p* < 0.0001; 4 days postinfection, F_3,4_ = 962.1, *p* < 0.0001; IFN-β1: 2 days postinfection, F_3,4_ = 46.39, *p* < 0.0014; 3 days postinfection, F_3,4_ = 804.6, *p* < 0.0001; 4 days postinfection, F_3,4_ = 31.04, *p* < 0.0031; IFN-λ1: 2 days postinfection, F_3,4_ = 670.4, *p* < 0.0001; 3 days postinfection, F_3,4_ = 472.7, *p* < 0.0001; 4 days postinfection, F_3,4_ = 810.5, *p* < 0.0001; * *p* < 0.05, ** *p* < 0.01, and **** *p* < 0.0001 vs. HCoV-OC43 at the same day postinfection). Data are presented as means ± SEMs and represent three independent experiments.

To determine whether auraptene induced antiviral interferon genes (IFN-α1, IFN-β1, and IFN-λ1), their expression level was evaluated using qRT-PCR. HCoV-OC43 infection induced IFN-α1, IFN-β1, and IFN-λ1 in MRC-5 cells time-dependently at 1, 2, 3, and 4 days postinfection. However, treatment with 4, 7, or 10 μM auraptene did not increase the expression level of these antiviral interferon genes in HCoV-OC43-infected cells (Figure 4). These data suggested that auraptene treatment itself did not induce antiviral IFN genes; instead, their expression was decreased due to the lower viral RNA level in auraptene-treated MRC-5 cells during HCoV-OC43 infection, as shown in Figure 2A. Therefore, auraptene protected HCoV-OC43-infected MRC-5 cells but not through the induction of the antiviral IFN genes, IFN-α1, IFN-β1, and IFN-λ1.

### 3.5. Auraptene Decreased MMP-9 mRNA Levels, which Increased with HCoV-OC43 Infection in MRC-5 Cells

**Figure 5:**
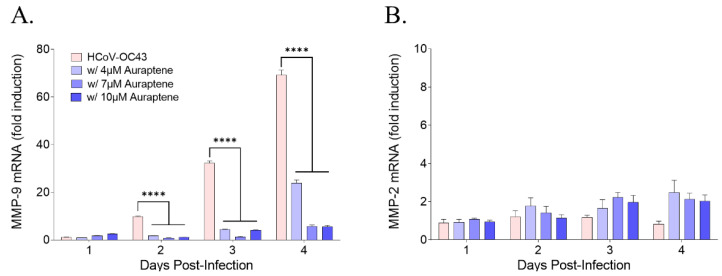
Auraptene treatment decreased the induced MMP-9 expression by HCoV-OC43 infection. (**A**,**B**) Level of MMP-9 (**A**) and MMP-2 (**B**) mRNA quantified in HCoV-OC43-infected MRC-5 cells treated with vehicle (pink bar) or 4, 7, or 10 μM auraptene (blue bar) using qRT-PCR at 1, 2, 3, and 4 days postinfection. Data were normalized to β-actin gene expression level. Data were analyzed using one-way ANOVA followed by Bonferroni’s multiple comparisons test (MMP-9: 2 days postinfection, F_3,4_ = 1093, *p* < 0.0001; 3 days postinfection, F_3,4_ = 811.7, *p* < 0.0001; 4 days postinfection, F_3,4_ = 527.3, *p* < 0.0001; **** *p* < 0.0001 vs. HCoV-OC43 at the same day postinfection). Data are presented as means ± SEMs and represent three independent experiments.

Auraptene has been reported to suppress matrix metalloproteinase (MMP)-2, MMP-7, and MMP-9 expression in human colon cancer cells [17], as well as MMP-2 and MMP-9 activity, to inhibit the migration and invasion of cancer cells [18]. Thus, we examined whether HCoV-OC43 infection induced MMP-2 and MMP-9 mRNA expression using qRT-PCR at 1, 2, 3, and 4 days postinfection. HCoV-OC43 infection increased MMP-9 mRNA expression in a time-dependent manner but not MMP-2 (Figure 5). Moreover, when administering auraptene at 4, 7, or 10 μM during HCoV-OC43 infection, MMP-9 mRNA expression was reduced in a dose-dependent manner at 4 days postinfection compared to virus-infected MRC-5 cells. These data showed that HCoV-OC43 infection induced MMP-9 but not MMP-2 mRNA expression in MRC-5 cells, and that MMP-9 mRNA expression was reduced in auraptene-treated MRC-5 cells.

## 4. Discussion

Auraptene is the most abundant prenyloxycoumarin in nature and commonly exists in the fruits of *Citrus* spp. [9]. Auraptene exhibits a variety of pharmacological and therapeutic properties, mediating host signaling pathways and inhibiting the functions of target proteins [10]. In this study, we showed auraptene’s antiviral activity against HCoV-OC43 infection in human lung fibroblast cells, MRC-5, by effectively inhibiting virus-induced cytopathic effects and the expression level of viral RNA and viral proteins during HCoV-OC43 infection.

Previously, auraptene was shown to have antiviral activity against influenza virus A, H1N1 [13]. Although auraptene has previously been shown to inhibit enterovirus 71 infection at the attachment and entry step of the viral life cycle by targeting capsid proteins, VP1 and VP2, using a reverse genetic system [14], the time-of-addition assay of auraptene against HCoV-OC43 in our study showed that auraptene inhibited HCoV-OC43 infection at the post-entry stage. These results suggest that auraptene exhibits distinct modes of action in different viral infections.

Among the well-known modes of action of auraptene as an anti-tumor agent, it suppressed the expression of MMP-2, MMP-7, and MMP-9 proteins in colon cancer cells and colonic mucosa from colitis mice [17] and also repressed MMP-2 and -9 activity to inhibit the migration and invasion of cervical and ovarian cancer cells [18]. Moreover, auraptene inhibited the *Porphyromonas gingivalis* bacterial infection which causes periodontal diseases by reducing the secretion and activity of MMP-8 and MMP-9 [19]. Similar to these previous studies, we found that auraptene treatment suppressed MMP-9 expression in HCoV-OC43-infected MRC-5 cells, and this suppression occurred in a dose-dependent manner, despite the induction of MMP-9 by HCoV-OC43 infection in a time-dependent manner.

In our study, we showed for the first time that HCoV-OC43 infection induced MMP-9 but not MMP-2 mRNA expression in MRC-5 cells. Virus infections have been shown to modulate MMP-9 expression. For instance, MMP-9 protein was increased by West Nile virus infection in the circulation and brain and was involved in its entry into the central nervous system (CNS) [20]. Similarly, human immunodeficiency virus Tat protein was found to upregulate MMP-9 expression, which can accelerate the trafficking of leukocytes into the CNS, resulting in the acquired immune deficiency syndrome dementia complex [21]. Dengue virus and Japanese encephalitis virus were both found to increase MMP-2 and MMP-9 expression and increase blood-brain barrier permeability, resulting in brain damage [22,23]. The upregulated MMP9 expression by Zika virus promotes virus entry into the testes by disrupting the blood–testis barrier [24]. Epstein–Barr virus oncoprotein induces MMP-9 expression, which may contribute to tumor invasion and metastasis [25].

In addition, MMP-9 is reported to modulate virus replication. Respiratory syncytial virus (RSV) infection induces MMP-9 expression in vitro and in vivo, whereas MMP-9 deletion decreases RSV replication [26]; however, MMP-9 also recruited neutrophiles resulting in the RSV clearance as antiviral activity in vivo [27]. A higher level of MMP-9 has been reported in influenza A virus-infected murine lung, whereas virus-infected MMP-9 deficient mice showed lower viral titer, being protected from lung disease by an effective immune response [28]. These data indicate that MMP-9 could be involved in HCoV-OC43 infection and replication. Moreover, Hepatitis B virus infection induces MMP-9 expression in immune cells, thereby evading host immunity by binding MMP-9 to IFN receptor I and blocking IFN signaling. This mechanism enables the virus to maintain a persistent infection [29]. Based on these results, it is possible that HCoV-OC43 infection also induces the expression of type I interferon genes, but the virus-induced MMP-9 proteins could potentially block the IFN signal, thereby evading the host’s antiviral response in MRC-5 cells, which could be blocked by auraptene treatment. However, to validate this hypothesis, further studies are required.

Recently, it was reported that SARS-CoV-2 infection induced MMP proteins. SARS-CoV-2 infection in K18-hACE2-transgenic mice (K18-hACE2) increased the expression of MMP-8, MMP-9, and MMP-14 proteins in the lung, suggesting that SARS-CoV-2 infection is associated with the activity of the MMP family which may cause tissue damage [30]. Furthermore, MMP-9 levels increased while MMP-2 levels decreased in COVID-19 patients, which is consistent with HCoV-OC43 infection in MRC-5 cells. Moreover, the increased serum MMP proteins levels in COVID-19 patients may be useful biomarkers of severe COVID-19 [31,32]. Therefore, HCoV-OC43 infection may induce the expression of MMP-9 proteins, which can modulate virus replication and pathophysiology. However, the treatment with auraptene has the potential to inhibit these effects, making it a promising therapeutic agent.

## 5. Conclusions

In this study, we presented the antiviral effect of a natural prenyloxycoumarin, auraptene, against human coronavirus HCoV-OC43 infection by inhibiting virus-induced cytopathic effects, viral RNA replication, and viral protein expression. A time-of-addition assay suggested that auraptene inhibited HCoV-OC43 infection at the post-entry stage of the virus life cycle. Furthermore, auraptene decreased the induced MMP-9 expression caused by HCoV-OC43 infection in human lung fibroblast cells. Thus, auraptene could be a future and potential anticoronaviral therapeutic agent. Further research is needed to clarify the molecular mechanism of auraptene in detail to allow its development into a therapeutic treatment with an excellent safety profile.

## Data Availability

Not applicable.
